# Organization of the human fetal subpallium

**DOI:** 10.3389/fnana.2013.00054

**Published:** 2014-01-16

**Authors:** Marie-Christin Pauly, Máté D. Döbrössy, Guido Nikkhah, Christian Winkler, Tobias Piroth

**Affiliations:** ^1^Department of Neurology, University Freiburg – Medical CenterFreiburg, Germany; ^2^Department of Stereotactic and Functional Neurosurgery, University Freiburg – Medical CenterFreiburg, Germany; ^3^Department of Neurosurgery, University Clinic ErlangenErlangen, Germany; ^4^Department of Neurology, Lindenbrunn HospitalCoppenbrügge, Germany

**Keywords:** subpallium, ganglionic eminence, striatum, development, patterning

## Abstract

The subpallium comprises large parts of the basal ganglia including striatum and globus pallidus. Genes and factors involved in the development of the subpallium have been extensively studied in most vertebrates, including amphibians, birds, and rodents. However, our knowledge on patterning of the human subpallium remains insufficient. Using double fluorescent immunohistochemistry, we investigated the protein distribution of transcription factors involved in patterning of the subventricular zone (SVZ) in the human forebrain at late embryonic development. Furthermore, we compared the development of cortical and striatal precursors between human fetal brain and E14 and E16 fetal rat brains. Our results reveal that DLX2 marks SVZ precursors in the entire subpallium. Individual subpallial subdomains can be identified based on co-expression of DLX2 with either PAX6 or NKX2-1. SVZ precursors in the dorsal LGE and preopto-hypothalamic boundary are characterized by DLX2/PAX6 co-expression, while precursors in the MGE and preoptic region co-express DLX2/NKX2-1. SVZ precursors in the ventral LGE are DLX2(+)/PAX6(-)/NKX2-1(-). In terms of staging comparisons, the development of the corpus striatum in the human fetal brain during late embryonic stages corresponds well with the development of the striatum observed in E14 fetal rat brains. Our study demonstrates that the pattern underlying the development of the subpallium is highly conserved between rodents and humans and suggests a similar function for these factors in human brain development. Moreover, our data directly influence the application of ganglionic eminence derived human tissue for cell therapeutic approaches in neurodegenerative disorders such as Huntington's disease.

## INTRODUCTION

During embryonic development, the future forebrain is already determined by expression of OTX2 in the neuroectoderm at the gastrula stage (Simeone et al., 1993). During the following neurulation the central nervous system – the so-called neural tube – forms by budding off from the ectoderm. The neural tube is divided into four longitudinal zones along the dorso-ventral axis (floor, basal, alar, and roof plate). Furthermore, the anterior part of the neural tube that will develop into the encephalon is divided into initially three transverse zones along the anterior-posterior axis: the anterior prosencephalon (forebrain), the mesencephalon (midbrain), and the posterior rhombencephalon (hindbrain). Prosencephalon and rhombencephalon will further divide into subdomains while the mesencephalon does not subdivide.

Our knowledge of mechanisms involved in controlling neural progenitor region patterning and brain development is almost completely derived from animal models. In contrast to earlier distinctions which were solely based on anatomic landmarks, the current widely accepted model of the prosencephalic subdivision provided by (Puelles and Rubenstein, 2003) relies on gene expression patterns of transcription factors (mainly of DLX, NKX, LHX, and PAX family members) in subventricular zone (SVZ) precursors of the particular area. According to this model, the prosencephalon is divided into the secondary prosencephalon (rostral) which represents the entire prechordal portion of the neural tube and into the caudal diencephalon (Puelles and Rubenstein, 2003) The caudal diencephalon is further subdivided into three so-called prosomeres (p1–p3) which will develop into habenular, thalamus, and prethalamus (PTh; Martinez-Ferre and Martinez, 2012). The secondary prosencephalon comprises the rostral diencephalon (hypothalamus) and the entire telencephalon. It is further divided into an evaginated and a non-evaginated domain (Puelles and Rubenstein, 2003). The evaginated secondary prosencephalon comprises the pallium, the lateral, and medial division of the ganglionic eminence (LGE respectively, MGE) and furthermore the optic vesicles. The pallium will develop into cortex and large parts of the limbic system, while LGE and MGE will give rise to the basal ganglia [striatum and globus pallidus (GPe)]. The ventral telencephalon (subpallium) and pallium are separated by a longitudinal boundary – the pallial-subpallial boundary (PSB) – which proceeds dorsally of the LGE. In addition to the evaginated secondary prosencephalon, the non-evaginated secondary prosencephalon comprises the telencephalic stalk and the hypothalamus [except the optic stalk (os)]. The telencephalic stalk includes the preoptic region (PO) and the preopto-hypothalamic boundary (POH). The POH is the boundary that separates subpallium and hypothalamus (Flames et al., 2007). The hypothalamus, in turn, is further subdivided into the rostral (alar) and a caudal (basal) domain.

Comparative expression analyses revealed that the prosomeric subdivision is conserved among all vertebrates (Moreno et al., 2009; Moreno and González, 2011). However, our knowledge on the genoarchitecture of the human prosencephalon remains insufficient. Various studies described the expression transcription factors involved in telencephalic patterning in the human fetal brain, but these studies mainly focused on corticogenesis and development of cortical interneurons (Terzić and Saraga-Babić, 1999; Lindsay et al., 2005; Bystron et al., 2006; Métin et al., 2007; Jakovcevski et al., 2011). However, systematic analyses regarding subdivisions and their boundaries are missing. In the present study, the protein expression of markers determining the secondary prosencephalon were analyzed in human fetuses at late embryonic stages (around 50–54 days post fertilization, dpf) and E14.0 and E16.0 rat fetuses were taken as reference. Our results show that the protein expression patterns of regional restricted transcription factors in the developing brain are conserved between humans and rodents. Furthermore our results show that the neuronal differentiation dynamics differ between rodents and humans.

## MATERIALS AND METHODS

### TISSUE COLLECTION

All experiments comprising human fetal tissue samples were in agreement with the German law. Experiments with human tissue samples were approved by the ethical committee of the University Freiburg. Human fetal tissue from routine elective abortions was collected with approved consent of the donating pregnant woman within the Multicentric Intracerebral Grafting in Huntington’s disease (MIG-HD) clinical transplantation program (ClinicalTrials.gov Identifier: NCT00190450). This paneuropean trial aims at intrastriatal transplantation of human fetal ganglionic eminence derived progenitor cells for the treatment of Huntington’s disease (HD). Tissue used in the present study was obtained in cases where a clinical application was not possible. Fetal tissue samples were collected in Hank’s balanced salt solution (HBSS) without Mg^2^^+^, Ca^2^^+^, or Dulbecco’s modified Eagle medium (DMEM; all purchased from Life Technologies, Grand Island, NY, USA) medium and stored at 4°C. The age of the fetuses was assessed morphometrically as described by Evtouchenko and collaborators (Evtouchenko et al., 1996). Evaluation of the health status of the pregnant woman was routinely performed by serological sample analysis for the human immune deficiency virus (HIV), hepatitis virus type B and C (HBV and HCV) and toxoplasmosis. For this study, brains from three human fetuses of 50–54 dpf measuring 23.5, 28.0, and 28.5 mm CRL were analyzed; two more human fetal brains of earlier developmental stages were examined beforehand to test the specificity of the antibody staining.

All animal experiments were approved by the local authorities beforehand. Male and female Sprague–Dawley rats were purchased from Charles River Laboratories (Sulzfeld, Germany) and housed in the animal facility of the University Freiburg Medical Center. Animals were kept under conventional housing conditions. The day after mating was considered as embryonic day 0 (E0). At the desired embryonic age, pregnant dams were sacrificed by i. p. injection of 150 mg/kg ketamine (Bayer, Leverkusen, Germany) and 15 mg/kg xylazine (Rompun®; Bayer) and additional cardiotomy. The abdomen of the animal was disinfected using 70% ethanol, and abdominal muscles were opened. The uterus was dissected and rinsed in sterile phosphate-buffered saline (PBS). Embryos were removed from the uterus and stored in HBSS or DMEM. The litter sizes usually ranged between 12 and 15 fetuses per dam. CRL of the fetuses was measured under a stereomicroscope and the age of the fetuses was calculated using embryonic staging data by Torres et al. (2008).

### IMMUNOHISTOCHEMISTRY

Fresh tissue samples from fetal CNS were snap frozen in Tissue-Tek O.C.T. compound (Sakura, Leiden, the Netherlands) in methyl butane (Merck, Darmstadt, Germany) that was cooled in a mixture of dry ice and ethanol. For human tissue samples, the CNS was dissected prior to snap freezing, whereas rat fetuses were completely frozen. Tissue was sectioned on a cryostat in coronal direction at 12 μm in eight series. Sections were mounted on Superfrost plus slides (R. Langenbrinck, Emmendingen, Germany). Fresh frozen sections were dried at 37°C for 20–30 min. For transcription factor staining, sections were fixed in acetone pre-cooled on dry ice for 10 min followed by extensive drying for 1 h. For DARPP32 staining, sections were fixed in 4% paraformaldehyde (PFA; Merck) in PBS. To prevent leakage of the antibody solution, glass slides were circulated with a silicone pen (Dako, Hamburg Germany). Sections were washed three times in PBS/0.1% Tween-20 (Calbiochem, Bad Soden, Germany; PBST), and blocked in PBS/0.2% Triton X-100 (Sigma Aldrich)/10% serum, species specific to the host of the secondary antibody. Primary antibody was diluted in antibody solution containing PBS/0.2% Triton X-100/2% serum for 2 h at RT, or overnight at 4°C. Primary antibodies and their specific epitopes, as well as the dilution and product information are listed in **Table 1**. The specificity of the antibodies has been demonstrated by the manufacturer and all antibodies are recommended to detect their specific target of human and rodent origin. However, we performed a protein sequence BLAST analysis (blastp) to provide the degree of homology to the human and rat protein. Where possible primary antibodies directed against the human protein were used. After incubation, the primary antibody solution was washed off, and secondary antibodies conjugated with either Alexa-488 or Alexa-568 fluorescent dyes (all obtained from Life Technologies) including DAPI as nuclear counter stain diluted in antibody solution were incubated for 2 h at RT. Afterwards, sections were washed three times in PBS/0.1% Tween-20. Acetone-fixed slides were post-fixed in 4% PFA (Merck). Sections were mounted with fluorescent mounting medium (Dako) and stored at 4°C in the dark. Controls including no-primary antibody have been performed to provide evidence of the antibody specificity.

**Table 1:** Antibodies validated to work on human and rat fetal tissue samples.

Antibody	Distributor (ordering information)	Dilution	Epitope	Homology to human, %	Homology to rat, %
Anti-DARPP32	Santa Cruz (sc-11365)	1:100	Amino acid 134–196 of bovine DARPP32	84	78
Anti-DLX2	Santa Cruz (sc-18140)	1:50	C-terminus of human DLX2	100	93
Anti-FOXP1	abcam (ab16645)	1:500	Amino acid 650 to C-terminus of human FOXP1	100	100
Anti-NKX2-1	Santa Cruz (sc-13040)	1:200	Amino acid 1–190 of human NKX2-1	100	99
Anti-NKX2-2	Developmental studies hybridoma bank	3 μg/ml	Chick NKX2-2	88	88
	Developmental studies hybridoma bank	3 μg/ml	Amino acid 1–223 of chick PAX6	99	92
Anti-PAX6	Santa Cruz (sc-32766)	1:100	Amino acid 1–206 of human PAX6	100	99
Anti-SATB2	abcam (ab34735)	1:250	Amino acid 700–733 of mouse SATB2	97	85

### CRESYL VIOLET STAINING

One whole series was stained with a standard cresyl violet staining. Briefly, sections were dried, fixed in 4% PFA in PBS, rinsed in water and incubated in 0,1% cresyl violet acetate solution at 37°C for 20 min. Afterwards, sections were again rinsed in water, dehydrated in ethanol, cleared in xylol and mounted with histofluid mounting medium (Marienfeld-Superior, Lauda-Königshofen, Germany). Cresyl violet stained series were used as reference for brain morphology.

### MICROSCOPY AND IMAGE COMPOSITION

Immunohistochemical sections were analyzed using an AX70 microscope (Olympus, Tokyo, Japan) that was equipped with a CC-12 camera and CellP imaging software (Olympus). Independent replicates of minimum three different donors were analyzed for all experiments. Representative single and double immune fluorescence stained sections were photographed with a 4× objective [numerical aperture (NA) 0.13] for human and E16 rat, a 10× objective (NA 0.3) for E14 rat, a 20× objective (NA 0.5) for details and a 100× (NA 1.3 oil immersion) objective for single cells. Cresyl violet stained sections were photographed using a 1.25×(NA 0.04) light microscopy objective. For images of whole sections, individual images photographed with a 10× objective were imported to Illustrator CS6 (Adobe Systems, San José, CA, USA) sequentially and aligned manually. The DAPI image was composed first; next, the file association was changed to the corresponding red or green fluorescent image. Histogram-controlled linear level adjustment, brightness and contrast adaptation, and cropping were performed using Photoshop CS6 (Adobe Systems). Images of brain areas without specific antibody signal were used as control for image adjustment. Figures were composed with Illustrator CS6 (Adobe Systems). The nomenclature of the fetal brain anatomy follows that used in previous studies on mammalian prosencephalon mapping (Puelles and Rubenstein, 2003; Flames et al., 2007; Moreno et al., 2009).

## RESULTS

**FIGURE 1:**
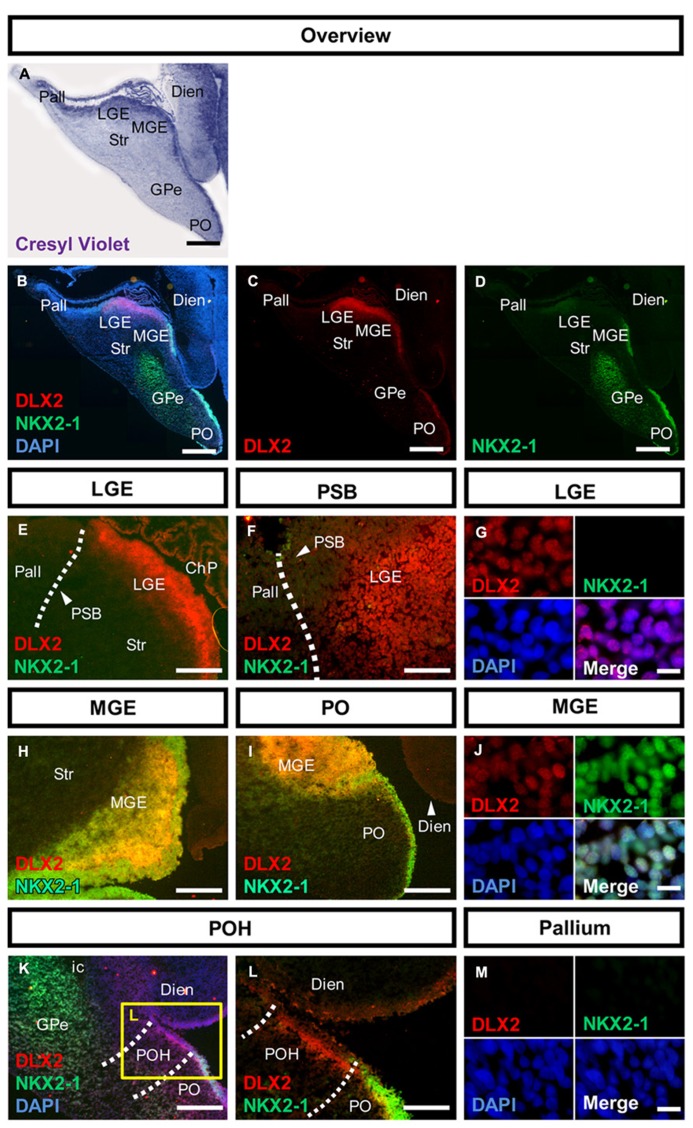
**Molecular characterization of the human subpallium.** **(A)** Photomicrographs of cresyl-violet coronal human subpallial sections. **(B–D)** Photomicrographs of sections stained against DLX2 (red) and NKX2-1 (green) and DAPI (blue). **(E,F)** DLX2-positive cells were detected in the LGE and end abruptly at the PSB. **(G)** Verification of nuclear DLX2 signal in the LGE and absence of NKX2-1. **(H–J)** DLX2/NKX2-1 co-expressing cells were detected in MGE and PO. **(K,L)** Cells within the boundary between subpallium and hypothalamus express DLX2 and lack NKX2-1. **(M)** Neither DLX2-positive nor NKX2-1 positive cells were detected in the pallium. Abbreviations: CGE, caudal ganglionic eminence; ChP, choroid plexus; DARPP32, dopamine- and cAMP-regulated phosphoprotein, 32 kDa; Dien, diencephalon; dLGE, dorsal part of the lateral ganglionic eminence; DLX2, distal-less homeobox protein 2; FOXP1, forkhead box protein P1; GPe, external segment of the globus pallidus; LGE, lateral ganglionic eminence; LV, lateral ventricle; MGE, medial ganglionic eminence; NKX2-1, homeobox protein NK-2 homolog A; NKX2-2, homeobox protein NK-2 homolog B; os, optic stalk; Pall, pallium; PAX6, paired box protein 6; PO, preoptic area; POH, preopto-hypothalamic boundary; PSB, pallial-subpallial boundary; PTh, prethalamus; PVN, paraventricular nucleus of the hypothalamus; SATB2, special AT-rich sequence-binding protein 2; SP, cortical subplate; SPV, supraopto-paraventricular region of the hypothalamus; Str, striatum; vLGE, ventral part of the lateral ganglionic eminence; ZLI, zona limitans intrathalamica. Scale bars: 1 mm in **A–D**. 500 μm in **E,H,I,K**; 200 μm in **L**; 100 μm in **F**; 10 μm in **G,J,M**.

**FIGURE 2:**
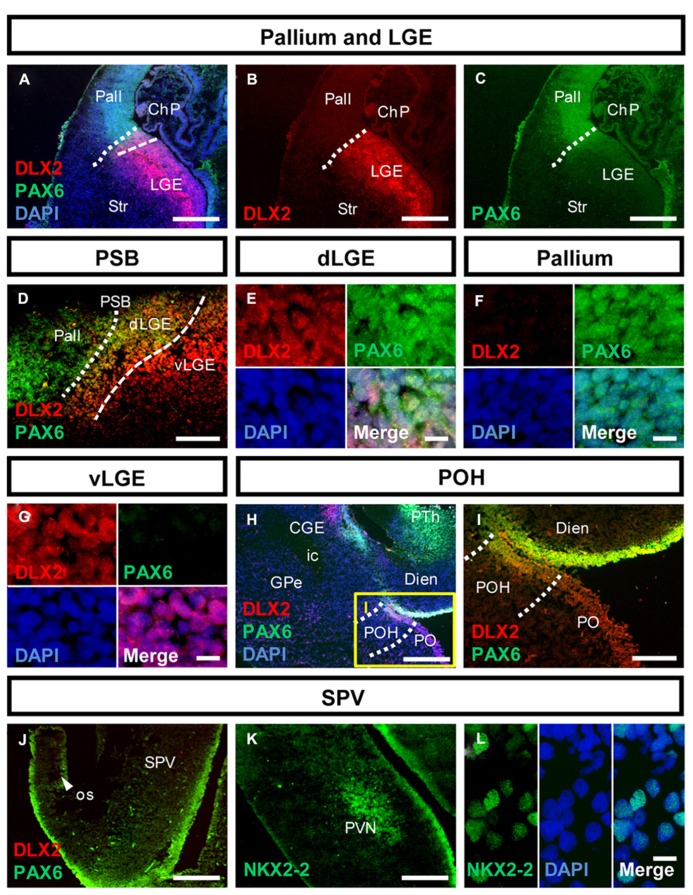
**Molecular identity of the pallial-subpallial boundary and the preopto-hypothalamic boundary in the human fetal brain.** **(A–C)** Photomicrographs of coronal human subpallial sections stained against DLX2 (red) and PAX6 (green); **(D)** the dLGE co-expresses DLX2 and PAX6. **(E–G)**: co-expressing DLX-2 positive and PAX6-positive cells were detected in the dLGE but not in the Pallium or LGE. **(H,I)** The boundary between subpallium and hypothalamus is characterized by DLX2/PAX6 co-expression **(J–L)** photomicrograph of the alar hypothalamus stained against **(J)** PAX6 (green) and DLX2 (red) and **(K,L)** NKX2-2 (green). **(J)** The alar hypothalamus is characterized by PAX6. **(K,L)** NKX2-2 is expressed in the SVZ of the AB boundary and in post-mitotic cells giving rise to the PVN. Abbreviations see **Figure 1**. Scale bars: 500 μm in **A–C,H,J,K**; 200 μm in **I**; 100 μm in **D**; 10 μm in **E–G,L**.

**FIGURE 3:**
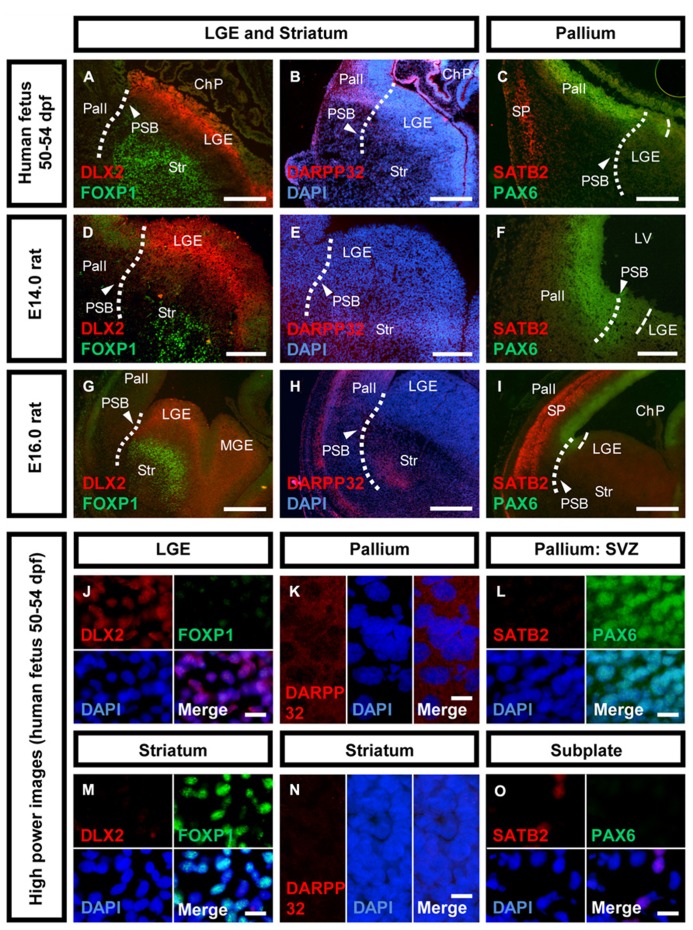
**Neuronal maturation of the striatum versus cortex in human and rat.**Photomicrographs of coronal sections of the human and rat telencephalon. **(A,D,G,J,M)** Expression of DLX2 (red) and FOXP1 (green) in the human, E14.0 and E16.0 rat LGE and striatum. **(B,E,H,K,N)** Expression of DARPP32 (red) and the nuclear counter stain DAPI (blue) in the human, E14.0 and E16.0 rat pallium, and striatum. **(C,F,I,L,O)** Expression of SATB2 (red) and PAX6 (green) in the human, E14.0 and E16.0 rat pallium, and cortical subplate. Abbreviations see **Figure 1**. Scale bars: 500 μm in **A–C,G–I**; 200 μm in **D–F**; 10 μm in **J–O**.

**FIGURE 4:**
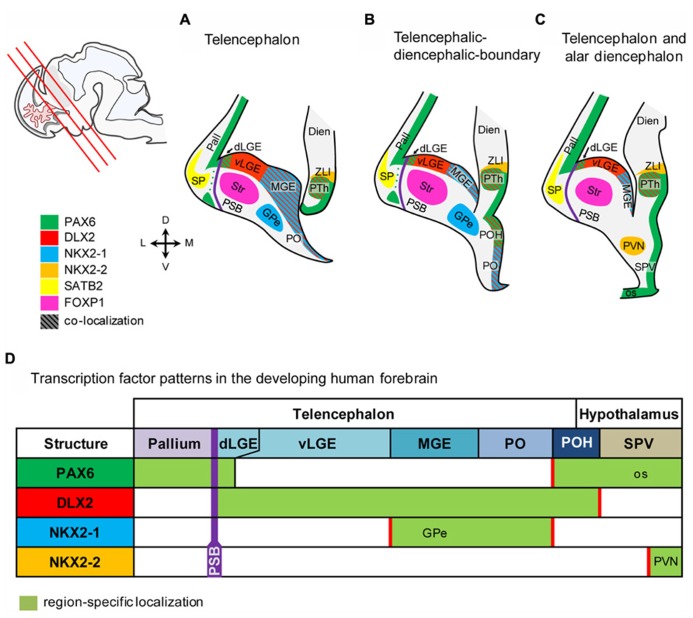
**Schematic representation of the marker expression in the human fetal forebrain.** **(A–C)** Schematic coronal sections of the telenecephalon, and alar dienecephalon. Section planes are indicated by red lines in the localizer on the left side. **(D)** Transcription factors expressed in subventricular precursors define molecular boundaries in the fetal human brain. Abbreviations see **Figure 1**.

The genoarchitecture of the subpallium as well as its dorsal and caudal boundaries was analyzed by co-immunohistochemistry of 50–54 dpf human fetuses (**Figures 1,2**). This time point represents the late embryonic stage of development (O’Rahilly and Müller, 2006). An overview of a typical coronal section of the subpallium stained with cresyl violet is provided in **Figure 1A**. Furthermore, we aimed to correlate neurogenesis in human fetuses at that stage with the development of the rat. Therefore, we compared the expression of mitotic and post-mitotic precursor markers in the striatal and cortical anlage between human fetuses and rat fetuses derived from embryonic day E14.0 and E16.0 (**Figure 3**). A schematic representation of our results is presented in **Figure 4**.

### DLX2 AND NKX2-1 CHARACTERIZE SUBPALLIAL REGIONS IN THE HUMAN FETAL BRAIN

In the human fetal subpallium, DLX2-positive cells were detected in the SVZ of LGE, MGE, PO, and POH (**Figure 1**). In the ganglionic eminence, DLX2-positive cells were detected in a stripe at the ventricular portion of the SVZ in the LGE, ending abrupt at the PSB (**Figures 1B,C,E,F** dotted line, **Figure 1G**). While NKX2-1-positive cells were not observed in the LGE, MGE, and PO are characterized by DLX2/NKX2-1 co-expression (**Figures 1B,D,H–J**). Post-mitotic expression of NKX2-1 was detected in the external segment of the GPe (**Figure 1K**). In turn, DLX2/NKX2-1 co-expressing cells were not detected in the POH (**Figures 1K,L**). Coincidently with the disappearance of NKX2-1 domain, the fusion of the anterior diencephalon at the telencephalic-diencephalic boundary was observed, indicating that in the human fetus, the boundary between subpallium and hypothalamus is not only morphologically, but also molecularly defined. In turn, no DLX2-positive or NKX2-1 positive cells were detected in the pallium at this stage (**Figure 1M**).

### PAX6 AND DLX2 DETERMINE THE BOUNDARIES OF THE SUBPALLIUM IN THE HUMAN FETAL BRAIN

Analysis of PAX6 and DLX2 co-expression allowed determining regions adjacent to the subpallium (**Figure 2**): PAX6-positive cells were detected in the SVZ of the pallium (**Figures 2A,C,D,F**). Cells co-expressing PAX6/DLX2 were detected in the dorsal LGE (dLGE) but not in the ventral LGE (vLGE) where PAX6 positive cells were not observed (**Figures 2A–G**). Thus, PAX6 divides the human LGE into two distinct domains (**Figures 2A,D** dashed line). In turn, the position of the PSB is determined by the PAX6-positive/DLX2-negative domain (pallium) and the PAX6-positive/DLX2-positive (dLGE) domain (**Figures 2A–D**, dotted line). Caudally, at the boundary between subpallium and hypothalamus, PAX6/DLX2 co-expression was detected in the POH (**Figures 2H,I**). PAX6 expression was furthermore detected in the alar hypothalamus. Here, it was expressed in the supraopto-paraventricular region (SPV) and the evaginating os, as well as in the retina (**Figure 2J** and not shown). The transcription factor NKX2-2 is a target of the Shh morphogen and involved in establishing the alar-basal (AB) boundary (Pabst et al., 2000). In the human fetus, NKX2-2 expression was found in SVZ precursors along the hypothalamic AB boundary, from where numerous cells migrate outward to generate the paraventricular nucleus of the hypothalamus (PVN; **Figures 2K,L**).

### DIFFERENT MATURATION DYNAMICS IN HUMAN AND RAT

To analyze the maturation of telencephalic structures precursor markers and post-mitotic markers were analyzed in the human fetal brain and compared with rat fetuses at embryonic day E14.0 and E16.0 (**Figure 3**). Striatal precursors located in the mantle zone (MZ) of the human LGE stained positive for FOXP1 (**Figure 3A**). The lack of DLX2/FOXP1 co-expression suggests that these cells are post-mitotic juvenile neurons (**Figures 3J,M**). To determine the developmental stage of the striatum, the expression of DLX2 and FOXP1 was also analyzed in E14.0 and E16.0 rat fetuses (**Figures 3D,G**). Both, E14.0 and E16.0 ganglionic eminence stained positive for DLX2 in the SVZ and FOXP1 in the MZ, indicating the presence of mitotic precursors and juvenile striatal neurons. To analyze whether mature striatal neurons are present in the 50–54 dpf human fetus, we analyzed the expression of DARPP32 (**Figures 3B,E,H**). DARPP32-positive cells were detected in the human pallium, but not in the striatum (**Figures 3B,K,N**). In the E14.0 rat brain, DARPP32 expression was not detected in any telencephalic region (**Figure 3E**). In the E16.0 rat fetus, DARPP32-positive cells were detected in a small group of cells within the developing ventral telencephalon located at the outer margin of the future striatum as well as in a narrow band in the outer cortical layer (**Figure 3H**). Additionally, differences between human and rat development were observed in the fetal pallium. The expression of the cortical subplate (SP) marker SATB2 was observed in a domain dorsal of the PSB but it was not expressed throughout the dorsal telencephalon (**Figure 3C**). We did not observe cells co-expressing PAX6/SATB2 in the pallial SVZ or cortical SP (**Figures 3L,O**). The E14.0 pallium did not stain positive for SATB2 while PAX6 was highly expressed (**Figure 3F**). In contrast, in the E16.0 rat brain, SATB2-positive cells were detected in the upper layer cortex indicating a high number of cortical precursors (**Figure 3I**). The expression domain of SATB2 is much larger as observed in the human fetus and comprises the entire dorsal telencephalon. As in the E14.0 fetal rat brain, pallial SVZ precursors in the human fetal brain were positive for PAX6 although the expression was reduced compared to E14.0.

## DISCUSSION

In the present study, we provide evidence that the genoarchitecture of the human fetal forebrain shares many features with that of developing rodents. An overview of the results from the present study is shown in **Figure 4**. Our data further reveal that the progression of cortical and basal ganglia development differs between human and rodents.

The SVZ of the entire human subpallium is characterized by DLX2, whereas, in turn, PAX6 characterizes the SVZ of the human pallium and large parts of the rostral diencephalon. Subpallial subdomains can be further distinguished based on co-localization between DLX2/NKX2-1 and DLX2/PAX6, respectively. MGE and PO are characterized by the co-localization of DLX2 and NKX2-1, while the dorsal-most part of the LGE and the POH can be identified based on DLX2 and PAX6 co-localization as well as by the lack of NKX2-1 expression. The rostral-most hypothalamic domain called SPV is characterized by PAX6, Caudal and rostral hypothalamus is separated by the AB boundary which is characterized by NKX2-2, post-mitotic expression of NKX2-2 was also detected in the PVN.

### THE MAMMALIAN SUBPALLIUM

The genoarchitecture of the subpallium has been thoroughly studied in many vertebrates including mammals, birds, reptiles, amphibians, and fish (Cobos et al., 2001; Flames et al., 2007; Métin et al., 2007; Moreno et al., 2008). Phylogenetic comparisons revealed an evolutionary conserved blueprint for the basal ganglia and amygdaloid complex among jawed vertebrates (Moreno et al., 2009; Medina et al., 2011). Protein expression data from the present study reveals a conserved pattern of subpallial neurogenesis in the human fetal brain. The subpallium is characterized by high expression of distal-less related (DLX) transcription factors (Robinson et al., 1991; Bulfone et al., 1993; Price, 1993; Corbin et al., 2000; present study). The LGE differs from MGE and PO by lacking expression of NKX2-1 (Sussel et al., 1999; Xu et al., 2008; present study). The rodent LGE progenitor domain is further subdivided into a dorsal and ventral domain based on PAX6 expression (Stoykova et al., 2000; Tole et al., 2005; Flames et al., 2007). Progenitors from the PAX6-positive dLGE migrate rostral to the olfactory tubercle and ventral to the extended amygdala, whereas progenitors from the PAX6-negative vLGE generate the dorsal (caudate-putamen) and ventral striatum (accumbens nucleus; Yun et al., 2003; Tole et al., 2005; García-López et al., 2008). The MGE it characterized by high expression of NKX2-1. Similar to the LGE, the MGE gives rise to multiple neuron types in the forebrain (Sussel et al., 1999; Du et al., 2008; Xu et al., 2008). The main projection neurons generated by the MGE are GABAergic neurons of the GPe (Flandin et al., 2010; present results). The GPe neurons are generated early in development. Following GPe neurogenesis, the MGE serves as main source of GABAergic interneurons in the telencephalon (Hernández-Miranda et al., 2010). The PO expresses high levels of NKX2-1 and DLX2, too, but can be distinguished from the ganglionic eminence by high expression levels of Shh as well as the by the lack of Gsx2 expression (Flames et al., 2007; García-López et al., 2008; Bardet et al., 2010; present data). The PO gives rise to the lateral and medial portion of the preoptic nucleus and furthermore to the majority of forebrain cholinergic neurons (Ch1-4, striatal interneurons; Thal et al., 1992; Marin et al., 2000; Elshatory and Gan, 2008; García-López et al., 2008). The PO serves as source for forebrain oligodentrocytes due to the high expression of Shh (García-López et al., 2008). Due to its transcriptional profile, the POH is considered as independent subdomain residing in the non-evaginated secondary prosencephalon (Flames et al., 2007; Moreno and González, 2011). While the PO expresses DLX2 as well as NKX2-1, the POH lacks NKX2-1 expression and expresses PAX6 instead (Flames et al., 2007; present data). In the embryonic brain, the POH constitutes the boundary that separates the subpallium from the hypothalamus, but it remains still unknown which cell population is derived from the POH in the adult brain. Our results reveal that in the human fetus NKX2-2 was detected in SVZ progenitor cells of along the AB boundary and the PVN. Furthermore, we detected NKX2-2-positive cells in SVZ precursors along the zona limitans intrathalamica (ZLI) and in the dorsal part of the lateral geniculate nucleus as well as in the ventromedial hypothalamic nucleus (not shown).

### THE PALLIAL-SUBPALLIAL BOUNDARY

Data from the present study reveal that in the human brain the PSB proceeds dorsally of the expression domain DLX2 (separating the ventral pallium from the dLGE). While POH and AB boundary are rather gene expression domains than boundaries, the PSB is a true boundary. The PSB is the only segmental boundary that proceeds longitudinally and divides the telencephalon into a dorsal and ventral portion (Kiecker and Lumsden, 2012). The position of the PSB is determined by the mutual repression of PAX6 and GSX2 (a transcription factor which acts upstream of DLX2) since mutations in either of the two genes result in irregular PSB placement (Stoykova et al., 2000; Toresson et al., 2000; Yun et al., 2001; Carney et al., 2009; Cocas et al., 2011). The position of the PSB determines a local organizer referred as anti-hem assumed to counteract the cortical hem located at the dorsal margin of the pallium (Assimacopoulos et al., 2003; Hoch et al., 2009; Subramanian et al., 2009). Recently, the hem was described for human fetuses between 5 and 7 gestational weeks, whereas the anti-hem has not been identified in the human fetus, yet (Roy et al., 2013). Analysis of mouse PAX6 mutants (Sey-/-) which lack anti-hem signals reveal enhanced interneuron migration from the MGE to the pallium and abnormal corticothalamic projection. In the present study we observed migrating precursors derived from the PAX6-positive domain around the PSB (not shown) indicating that the anti-hem might also exist in the human fetal brain.

### MATURATION DYNAMICS IN THE HUMAN FOREBRAIN DIFFERS FROM RATS

The development of the human cortex has been object of intensive research during the past three decades (Meyer et al., 2000; Bystron et al., 2006; Bayatti et al., 2008; Clowry et al., 2010; Molnár and Clowry, 2012). Cortical neurogenesis is initiated by the appearance of Reelin-producing Cajal-Retzius cells derived from the cortical hem system from 5 gestational weeks onwards (Meyer et al., 2000; Bystron et al., 2006). Migrating neural precursors from the PAX6-positive SVZ migrate radially and divide the upper preplate into the outer marginal zone and the inner SP at 7–8 gestational weeks (Zecević, 1993; Meyer et al., 2000; Bystron et al., 2006). Our results show that SATB2-positive SP precursors were detected at 50 dpf in the human brain, which coincides with results from previous studies (Clowry et al., 2010).

In turn, the development of the basal ganglia in the human brain is less well characterized. In the rodent brain, the majority of LGE-derived precursors will give rise to striatal projection neurons (Nakao et al., 1996; Stenman et al., 2003). Results from the present study reveal that immature striatal neurons are characterized by FOXP1 expression and can be detected already at 50 dpf. In turn, post-mitotic cells derived from the MGE giving rise to projection neurons of the GPe can be identified by NXK2-1 expression. Regarding the development of the human striatum, Carri et al. (2013) describes the expression pattern of several telencephalic transcription factors in the human fetus of 11 gestational weeks. The authors detected expression of early telencephalic transcription factors such as GSX2, OTX2, and FOXG1 in the SVZ of the LGE and detected post-mitotic striatal marker FOXP1&2, CTIP2, and DARPP32 in the putamen and caudate nucleus at the same developmental stage (Carri et al., 2013). These data together with our results from the present study indicate that proliferation and neuronal maturation in the human striatum run in parallel during striatal neurogenesis.

### RELEVANCE OF STRIATAL DEVELOPMENT FOR THE TREATMENT OF HUNTINGTON’S DISEASE

Due to the pronounced loss of striatal projection neurons, HD has been targeted by cell therapeutic approaches (Rosser and Bachoud-Lévi, 2012). The concept of this therapy is the transplantation of ganglionic eminence precursor cells into the diseased striatum, where the cells replace degenerated striatal MSN and thereby reconstruct the damaged striatal output, mostly that into the GPe (Pauly et al., 2012; Trueman et al., 2012). Ganglionic eminence tissue derived from E13.0 to E16.0 rat fetuses grafted into a rat model of HD revealed that the best donor age lies around E14.0 which is coincides with the finding that cells for neural grafting must be obtained around the time point of peak neurogenesis (Olson et al., 1983; Watts et al., 2000; Schackel et al., 2013). Birth-dating experiments prior to and immediately after grafting revealed that striatal grafts are derived from both, dividing and post-mitotic precursor cells (Fricker-Gates et al., 2004). Our present data from human striatal development reveal that mitotic and post-mitotic precursors are present in the developing human ganglionic eminence during late embryonic stages, whereas mature DARPP32-positive MSN are missing.

For clinical transplantation trials on HD patients, donor tissue derived from 8 to 12 week old fetuses was mainly used (Kopyov et al., 1998; Bachoud-Lévi et al., 2000; Hauser et al., 2002; Rosser et al., 2002; Gallina et al., 2010, 2008). However, one of the unresolved questions is the optimal donor age for clinical transplantation (Freeman et al., 2011). The Carnegie Staging, typically consulted for developmental comparisons, favors the use of young donors (Carnegie Stage 19; 16–18.5 mm CRL; 43–46 dpf in humans; Streeter, 1942; O’Rahilly and Müller, 2006). However, our present data, as well as data from previous studies reveal that basal ganglia development in humans lasts over a longer period, thus rendering late embryonic stages more suitable for cell transplantation in HD (Clancy et al., 2001; Del Bigio, 2011; Carri et al., 2013).

## CONCLUSION

In this study, we showed that the expression of regional restricted transcription factors in the developing human and rodent brain reveals a conserved pattern between in both species. In turn, rodents and humans differ in their neuronal differentiation dynamics. In the human fetus, proliferating precursors and post-mitotic cells were detected to a high degree at the same time, indicating that these processes run in parallel during human brain development. In rat fetuses, proliferation and neuronal differentiation appear as sequential processes. These findings have direct influence on application of cell therapies for HD patients since the optimal donor age for grafting is not determined. The present study increases our understanding of forebrain development in humans and provides important new aspects for future cell therapy applications for HD.

## Conflict of Interest Statement

The authors declare that the research was conducted in the absence of any commercial or financial relationships that could be construed as a potential conflict of interest.
